# Insecticidal Paints: A Realistic Approach to Vector Control?

**DOI:** 10.1371/journal.pntd.0004518

**Published:** 2016-04-21

**Authors:** Karin L. Schiøler, Michael Alifrangis, Uriel Kitron, Flemming Konradsen

**Affiliations:** 1 Global Health Section, Department of Public Health, University of Copenhagen, Copenhagen, Denmark; 2 Centre for Medical Parasitology, Department of Immunology and Microbiology, University of Copenhagen, Copenhagen, Denmark; 3 Department of Infectious Diseases, Copenhagen University Hospital, Copenhagen, Denmark; 4 Department of Environmental Sciences, Emory University, Atlanta, Georgia, United States of America; 5 Fogarty International Center, National Institutes of Health, Bethesda, Maryland, United States of America; Hebrew University-Hadassah Medical School, ISRAEL

## Recoloring an Old Tool

Insecticidal paints (IPs) have been commercially available for several years, mainly in Europe and North America, where they are promoted against nuisance pests that dwell on walls and ceilings. Although IPs have been suggested for the control of disease vectors since the 1940s, the concept has never gained much attention compared with Indoor Residual Spraying (IRS), which offers the same basic mode of action. Today, however, IPs are receiving renewed interest for their potential use against disease vectors. This interest can be attributed to several factors, of which we list the most important below, along with the concerns that should be addressed before this intervention tool achieves widespread application.

## Novel Paint Technology

Recent advances in paint technology have guided the development of novel “ready-to-use” paint formulations in which microencapsulated insecticides, or active ingredients (AIs), are embedded in the paint matrix and gradually released on the surface of the dried paint. It is argued that the slow-release mechanisms enable uniform AI surface concentrations with prolonged residual effect, as compared to earlier IP products using a simple admixture of AIs in standard paint formulations. Depending on the chemical action of the embedded AIs, IPs may display different properties, including spatial repellency, contact irritancy, toxicity, insecticide synergy, or insect growth regulation. Importantly, different AIs can be combined in a single IP to ensure different modes of action in one product.

As for IRS, IPs can offer simultaneous protection across a wide range of vector-borne diseases (VBDs), including malaria and neglected tropical diseases (NTDs), such as Chagas, leishmaniasis, lymphatic filariasis, dengue, and chikungunya. However, IPs may be easily applied by homeowners, caretakers, and private contractors alike, thus eliminating the need for specially trained personnel and large-scale logistical planning as is required for IRS. Notably, slow-release IPs have been developed for both interior and exterior surfaces, extending their range of use beyond that of IRS. In fact, exterior IPs could be the preferred option for leishmaniasis control, as the phlebotomine vectors scale across exterior walls. Exterior IPs could also prove of interest in areas where existing indoor interventions have led endophilic vectors to adapt exophilic traits or have induced a shift in species dominance towards more exophilic vectors.

## Prolonged Residual Effect

A residual effect of up to 32 months against *Triatoma infestans* was recently demonstrated in an IP field trial in Bolivia [1], reinforcing previous findings of long-term impact of IPs on this particular NTD vector [2–4]. Laboratory- and field-based studies have so far demonstrated a residual effect of IPs of up to 12 months on different anopheline, culicine, and *Glossina* species [5–8]. Large-scale efficacy trials are still lacking for these and other important vectors, including aedine and phlebotomine species. However, the demonstrated efficacy of more than 2.5 years for triatomines implies IP performance comparable to that of long-lasting insecticide-treated nets (LLINs) and, notably, superior performance to that of IRS. Furthermore, with the exception of dichlorodiphenyltrichloroethane (DDT), all commonly used AIs require two or more annual IRS applications in areas with perennial disease transmission, suggesting that IPs may offer a cost-effective alternative to IRS, at least in terms of reapplication requirements. While documentation of IP vector impact is beginning to accumulate, evidence of disease reduction is still missing. A large-scale trial of the clinical impact of IPs on Chagas is reportedly in the initial stage [7]. However, at this point there is no documentation of afforded protection against VBDs by current IP products at the individual, household, or community level.

## Consumer-Driven Disease Prevention

Significant proportions of those who are poorest and most at risk of VBDs remain completely reliant on government-subsidized intervention programs. As such, many people may be restricted from being active consumers of IPs, given their insufficient purchasing power and unsuitable dwellings for IP application. Yet, socioeconomic development throughout disease-endemic areas of Asia, the Pacific, Africa, and the Americas is allowing private households, public institutions, and commercial enterprises to increasingly invest in vector control measures, such as coils, aerosol sprays, and fumigants, as well as insecticide-treated nets, curtains, and screens. The ability and willingness to pay for disease prevention is also reflected in a growing private sector offering vector control solutions, including IRS, to residential and commercial customers, especially in larger urban centers.

Consumer-driven disease prevention, as opposed to government-operated top-down programs, may have reached a crucial tipping point in many areas, suggesting a large-scale and so far uncontested market for IPs that offers both decorative (with choice of colors) and surface-protective properties in addition to vector control. It is argued that IPs could obtain universal availability relatively quickly, as the IP technology is easily adapted to the existing manufacturing, distribution, and sales networks for conventional paint. This critical incentive for commercial development, production, and marketing of IPs has already garnered the attention of multinational corporations, such as paint company AkzoNobel and insecticide producer Bayer. In 2013, AkzoNobel launched an IP production facility in West Africa and initiated development activities in India [9,10]. Last year, Bayer signed an agreement with the IP producer, Inesfly, for distribution rights to two of their main products [11].

## Rapid Urbanization and Changing Building Structures

Insecticidal paints are considered suitable for urban, peri-urban, and rural communities alike. However, they may prove particularly effective in transitory regions where expanding urbanization is changing the activity and distribution of several VBDs, including dengue, chikungunya, and leishmaniasis—and to some extent malaria [12–14]. Increased urbanization and general economic development are also creating new residential patterns and affecting building practices in most disease-endemic areas. This is noted by a shift towards modern building materials, including cement, plasters, plywood, and corrugated metal, which are far better suited for paint application than traditional materials, e.g., wattle and daub. Importantly, paint adhesion, application methods, and AI activity are certain to be influenced by specific properties of both modern and traditional materials (e.g., low pH of cement, high porosity of wattle and daub), implying that further studies are needed for IP manufacturers to adapt their product range to the predominant building materials of a given region.

## Expanding Geographical Distribution of Vector-Borne Disease

Introduction of vectors and/or pathogens into new areas of the world, such as *Aedes albopictus* into southern and central Europe; leishmania species into southern Europe, North America, and Australia; chikungunya and Zika viruses into the Americas; and, not least, the expansion and intensification of dengue virus transmission throughout the pan- and subtropical areas, is creating potential new markets for IPs. Notably, these are often markets where suitable building structures and consumer patterns for purchasing of conventional paints are already well established, suggesting rapid IP uptake by a strong consumer base using existing marketing channels. Given that IPs offer the same functions as conventional paints, i.e., the protection and decoration of building surfaces, user acceptance is likely to be high when compared to IRS, as the latter often causes blemishes and peels on the treated surfaces and is considered highly intrusive by many homeowners.

## Insecticide Resistance

Insecticide resistance, in the form of physiological, biochemical, and/or behavioral resistance, presents a major problem for current vector control efforts. Increased levels of pyrethroid resistance are particularly problematic, as this class of insecticides remains the predominant choice for IRS and is exclusively used for LLINs [15]. A notable feature of the IP technology is the ability to embed multiple AIs in the same IP. This includes the different classes of conventional insecticides as well as insecticide synergists and insect growth regulators. The combination of different AIs could overcome existing insecticide resistance and impede the development of new resistance traits. However, as the potency of embedded AIs fade over time, it is argued that IPs could prompt the development of cross-resistance if maintained inappropriately. Requirements for resistance management, including attestation of IP shelf life, quality control, and monitoring for regionally appropriate AIs, will therefore be a major regulatory challenge given the prospect of numerous production sites and commercial routes from outlet to end-user. The uncontrolled use of IPs stands in contrast to most IRS interventions, in which the top-down approach reduces the risk of specific AIs being propagated in areas with noted resistance.

## Policy Requirements for Addressing Health and Environmental Implications

The dependency of IPs on a group of chemical compounds that present potential health impacts on humans, as well as environmental hazards at the site of production, application, and disposal, could discourage the use of IPs as opposed to alternative and more eco-friendly vector control methods, such as source reduction and improved housing. Indeed, the human and ecological safety of current IPs should be comparable to that of existing insecticide-based interventions, given a restricted use of AIs approved by the World Health Organization’s Pesticide Evaluation Scheme (WHOPES). However, IPs may present a different safety profile given their slow-release mechanism and prolonged activity. Currently, safety approvals have only been achieved at national levels, while certification by the WHO and other international organizations are awaited. A central point of concern is the lack of a cohesive strategy for environmental management throughout all stages of the IP life cycle. The need for policy and guidance in terms of safe IP production, sale, application, and disposal of IP-related items (e.g., used brushes, canisters, and out-of-date products) is obvious, if a repeat of previous experiences with poor product stewardship of vector control interventions is to be avoided.

## Studies Required in Support of Policy and Guidelines Development

In the case of IPs, the conventional routes of product validation, dissemination, and end-of-life management are at high risk of being outpaced by commercial interests and end-user demands. To ensure that timely and relevant policies are based on adequate evidence, there is a need for high-quality and well-coordinated multicenter IP-studies addressing (i) human and ecological safety, (ii) vector and disease impact, (iii) appropriate AI combination strategies for areas with pre-existing insecticide resistance, (iv) economic assessment comparing IPs with alternative interventions, and (v) consumer acceptability and product applicability. We suggest that such studies and the rigorous assessment and synthesis of the generated data be planned and executed by the international research community in collaboration with international organizations and donors, national health authorities, and the commercial sector.

Regardless, IPs cannot deliver a substantial public health impact unless they are readily available to those who are most vulnerable to VBDs—the poor and largely rural populations. For this group, IPs may only serve as a complementary intervention to existing LLINs and IRS programs if substantially subsidized.
